# Chemical, microbial and antibiotic susceptibility analyses of groundwater after a major flood event in Chennai

**DOI:** 10.1038/sdata.2017.135

**Published:** 2017-10-10

**Authors:** Ganesan Gowrisankar, Ramachandran Chelliah, Sudha Rani Ramakrishnan, Vetrimurugan Elumalai, Saravanan Dhanamadhavan, Karthikeyan Brindha, Usha Antony, Lakshmanan Elango

**Affiliations:** 1 Department of Geology, Anna University, Sardar Patel Road, Guindy, Chennai 600025, India; 2 Centre for Food Technology, Department of Biotechnology, Anna University, Sardar Patel Road, Guindy, Chennai 600025, India; 3 Department of Hydrology, University of Zululand, Kwa-Dlangezwa 3886, South Africa; 4 Department of Civil and Environmental Engineering, National University of Singapore, Singapore 117576, Singapore

**Keywords:** Microbiology, Biogeochemistry

## Abstract

During floods, human exposure to pathogens through contaminated water leads to the outbreak of epidemic diseases. This research presents the first extensive assessment of surface and groundwater samples collected immediately after a flood (December 2015) and post-flood (April 2016) from the Adyar River of Chennai, a major city in India, for major ions, trace metals, bacterial population, and pathogens. Severe rains in a short period of time resulted in flooding which inundated the wells, allowing the entry of sewage contaminated river water into the groundwater zone. This has led to bacterial counts and chemical ions exceeding Bureau of Indian Standard’s recommended limits in most flood affected areas. Pathogens isolated from the groundwater showed resistance to antibiotics, namely ceftriaxone, doxycycline and nalidixic acid. However, they were sensitive to chloramphenicol, ciprofloxacin, norfloxacin, and tetracycline. Determining the antibiotic susceptibility of pathogens will help in the treatment of humans affected by contaminated water through an appropriate selection of prescribed medication.

## Background & Summary

Climate change in recent years is undeniable. Temperature variation has increased the atmospheric water vapor and subsequently the frequency of high rainfall events over the last few decades in many regions of the world^1^. Rainfall over an extended period before a high-intensity rainfall event leads to a rise in groundwater table and saturation of vadose zone, which is likely to exacerbate flooding^2^. Flash floods in Rye River, North Yorkshire (June 2005); Kosi River, Nepal, and India (June 2013); Mumbai, India (June 2015) and Mississippi River, USA (December 2015) have resulted in infrastructure damage and loss of human lives. Floods result in both positive and negative impacts on groundwater resources. A few researchers have studied the effects of the flood on groundwater in the recent past^3,4^. Floods will increase regional groundwater level^5^ and recharge of flood waters change the chemical and microbial constituents. The concentration of ions in groundwater decreases after floods^6^ and such changes in groundwater chemistry affect the biological activity in the aquifers^7^.

Floods are severe and common causes of epidemics in the low-income countries of Asia and Africa^8,9^. They increase the spread of water-borne diseases such as typhoid fever, cholera, leptospirosis and hepatitis A as well as vector-borne diseases namely malaria, dengue hemorrhagic fever, yellow fever and West Nile fever^9^. Waterborne pathogen contamination and related diseases are major concerns throughout the world^10^. In developing countries like India, a large number of economically weaker communities live on river banks of urban areas in small houses and huts. This population depends on groundwater for domestic purposes throughout the day, in the absence of a piped water supply^11^. People residing in or visiting such premises and consuming contaminated water from wells will be at risk of gastrointestinal problems associated with the infectious agents. The impact of flooding on groundwater quality in such regions will have a large bearing on human health as well as on the aquifer environment. The ingestion of bacteria such as *Salmonella typhi* and *Salmonella paratyphi* triggers the severity. These human restricted pathogens with no animal reservoir get transmitted through contaminated food, water or contact with fecal matter from infected individuals^12^.

It is essential to understand the impact of flooding on groundwater contamination by studying the pathogenicity and antibiotic susceptibility of microbes to draw a prognosis to diagnose effective antibiotics in associated health issues. No previous study has reported on the chemical and microbial quality of water affected by extreme floods as well as the virulence and antibiotic susceptibility of the thriving pathogens under the above circumstances. These studies are important to prevent the outbreak of epidemic diseases during calamities such as a flood. Hence, we studied the impact of an unprecedented flood that devastated the city of Chennai in India during December 2015.

**Figure 1:**
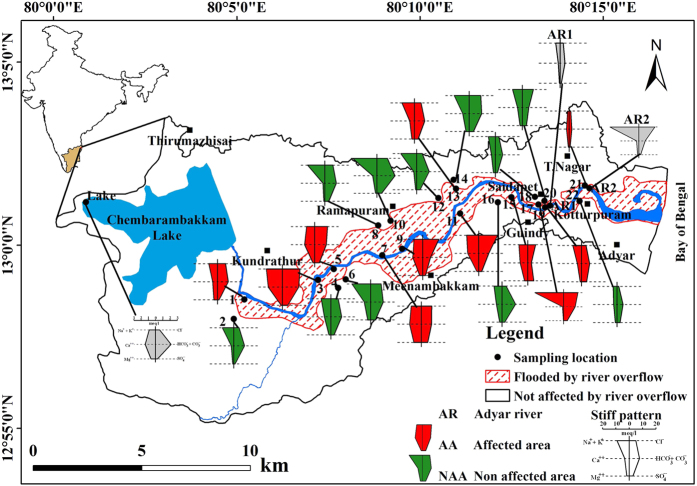


Continued heavy rainfall during the last fortnight of November 2015 and unprecedented rainfall on 1^st^ December 2015 in Adyar River (AR) catchment (Fig. 1), resulted in the inundation of a large area along the river banks on 2^nd^ December 2015. A total of 269 people died due to these floods^13^. These flood events led to scientific studies on urban flood management configuration^14^, implementation of climate adaptation strategies, revamping the existing infrastructure for unpredictable climatic conditions^15^ and increased micro plastic pellets along the coast^16^. These studies did not focus on chemical and microbial water quality as well as the risk posed to human health due to groundwater contamination. Further, no reports are available on microbial contamination of groundwater due to severe floods and antibiotic susceptibility. This is the first study that has aimed to assess the impact of severe floods on the chemical and microbial quality of groundwater and river water to determine the bacterial pathogenicity and *in vitro* antibiotic susceptibility.

## Methods

### Preparation of maps

The AR catchment (245.43 km^2^) is predominantly urbanized except for some restricted regions in the west that practice agriculture. As per the census in 2011, about 0.98 million people are living in this area. We assessed the water level in the river and inundated areas on 2^nd^ December 2015 by field visits carried out on the same day and on 30^th^ December 2015 by observation of the indications and marks on the buildings/walls as well as from information provided by the local community. We deciphered the extent of area flooded on either side of the river banks using the digital elevation model based on field observation (Fig. 1). We prepared maps using ArcMap v.10.1.

### Sampling

We decided the location of sampling wells to collect groundwater from during fieldwork on 31^st^ December. We performed it in a way such that there were no major contamination sources nearby, such as: dairy farms, garbage bins, and septic tanks. From eleven such sites (Fig. 1) along the AR, we selected a representative well from a flood inundated area and a corresponding well a few hundred meters away to represent the non-inundated area. In affected areas, the chosen wells were submerged under the flood water and hence the contaminated river water directly entered the wells. Surface water samples included the Chembarambakkam Lake (CL) and two locations in AR (upstream AR1 and downstream AR2, Fig. 1).

We collected samples after a flood (AF) on 31^st^ December 2015 and also three months later on 1^st^ April 2016 which represents post-flood (PF). Using a water level indicator (Solinist 101) we measured the groundwater level in the wells. During field visits, we collected three sets of water samples, among them two sets of samples (one for major ions and other for trace elements analyses) in clean polyethylene bottles of 500 ml capacity. We pre-cleaned these polyethylene bottles by washing them after soaking in 1:1 diluted HNO_3_ for 24 h and finally by glass distilled water. Further, we rinsed the bottles twice with the sample collected after filtering it using 0.22 μm filter paper (Merck, Mumbai). We acidified the samples collected for trace element analysis using HNO_3_. We collected another set of water samples for microbiological analysis in 50 ml autoclaved, screw-capped centrifuge tubes which were then stored at 4 °C to prevent changes in the microbial community after sampling and subjected to analysis within 18 h.

### Chemical analysis

We measured the electrical conductivity (EC) and pH of water samples in the field using the digital meter (EUREKA Sub 2 manta). We measured carbonate (CO_3_^2−^) and bicarbonate (HCO_3_^−^) at sampling sites with alkalinity test kits (Merck 1.11109.0001). We determined the concentration of the major ions (Calcium—Ca^2+^, Magnesium—Mg^2+^, Sodium—Na^+^, Potassium—K^+^, Chloride—Cl^−^ and Sulphate—SO_4_^2−^) by ion chromatography (Metrohm 850) and trace elements (Silica—Si, Silver—Ag, Aluminium—Al, Boron—B, Cadmium—Cd, Cobalt—Co, Chromium—Cr, Copper—Cu, Iron—Fe, Lithium—Li, Manganese—Mn, Nickel—Ni, Lead—Pb, Zinc—Zn and Phosphorus—P) by inductively coupled plasma-optical emission spectroscopy (Agilent 710 series). We ensured the accuracy of chemical analyses (Data Citation 1) by frequently measuring the concentration in standards, blanks as well as by calculation of the ion balance error (<±10%). Further, we compared the total weight of the measured ion with the total dissolved solids (TDS). We calculated the TDS from the formula, TDS=0.64×EC^17^. We plotted the stiff diagram to represent the chemistry of water samples using AquaChem v.4.0.

### Microbial analysis

We used spread plate (SP) technique on nutrient agar and incubation at 30 °C for 48 h^18^ to determine the total viable bacteria (log CFU ml^−1^). We analyzed samples in duplicates after serial dilution up to 10^−7^. Selective media used to isolate the water-borne pathogens included Xylose-Lysine Deoxycholate Agar for *Shigella*, Eosin Methylene Blue agar for *Enterobacter* and *Escherichia*, *Staphylococcus* agar for *Staphylococcus*, *Streptococcus* Agar for *Streptococcus*, Thiosulphate Citrate Bile Salts Sucrose agar for *Vibrio* and Rappaport-Vassiliadis Medium for *Salmonella* species. We isolated colonies from nutrient agar and selective media by sub-culturing twice to obtain single colonies. Individual isolates were then selected based on diverse morphological appearance so as to cover the entire range of microorganisms from the respective agar. We identified all isolates by Gram stain and also subjected them to other primary and secondary biochemical tests. We streaked the isolates on nutrient agar slants to prepare sample cultures for polymerase chain reaction amplification and to sequence their 16S rRNA genes for comparison with public and in-house databases. Reactions were set up with InstaGene Matrix (Bio-Rad, USA) and we performed the sequencing using Big Dye terminator cycle sequencing kit (Applied BioSystems, USA). We resolved the sequenced products on an automated DNA sequencing system (Applied BioSystems, model 3730XL, USA) and matched them in Basic Local Alignment Search Tool (BLAST) v.2.2.9. Further, we registered the sequences in GenBank (Data Citation 2), NCBI to obtain the accession numbers.

### Antibiotic susceptibility

We used antibiotics commonly prescribed for treatment in India at the recommended dosage to perform the antibiotic susceptibility test by disc-diffusion method^19^. Antibiotics used were Ceftriaxone—CTR 30 μg, Chloramphenicol—C 30 μg, Doxycycline—DO 30 μg, Tetracycline—TE 30 μg, Ciprofloxacin—CIP 5 μg, Norfloxacin—NX 10 μg and Nalidixic acid—NA 30 μg. From the results obtained we classified isolates as resistant or sensitive to a particular antibiotic (Data Citation 1) using reference values from the National Committee for Clinical Laboratory Standards, USA^20^.

## Data Records

The datasets are hosted in Figshare (Data Citation 1) and GenBank (Data Citation 2). The chemical and microbial data in Figshare contains the latitude, longitude, date of sampling, pH, EC, TDS, dissolved oxygen, calcium, magnesium, sodium, potassium, chloride, sulphate, bicarbonate, silica, silver, aluminium, boron, cadmium, cobalt, chromium, copper, iron, lithium, manganese, nickel, lead, zinc, phosphorus, total bacterial count, coliforms, *E.coli, E.aerogenes, S.epidermidis, S.flexneri, S.pyogenes, S.typhi, V.cholerae* for all locations as well as antibiotic susceptibility of bacterial isolates. The genotype data in GenBank contains sequence information (16S rRNA) for each pathogen isolated from the water samples.

## Technical Validation

### The flood event

Chennai is the capital city of Tamil Nadu state in India with an aerial extent of 1,189 km^2^. Elevation of the area varies from 0 m near the coast to 154 m along the southern boundary. Both tropical wet and dry climate prevail with humidity in the range of 65–85%. The weather report showed a maximum temperature up to 44 °C on some days between May and June and a minimum temperature of 17–28 °C between December and January. Annual rainfall is about 1,200 mm from the southwest monsoon (July to September), and northeast monsoon (October to December). On average, five depressions form in a year, primarily in the Bay of Bengal, and most of them result in high rainfall. A tributary originates from CL, joins with AR, flows eastwards through Chennai city (about 45 km) and drains into the Bay of Bengal. The urban population receives water from this lake after treatment. Sewage and solid waste discharged from domestic and industrial sources pollute the AR. In 2014, the groundwater quality around this river was reported^21^. Under normal conditions, AR is dry during the non-monsoon period in the upper reaches. However, further downstream, treated wastewater from a sewage treatment plant located near sampling well no. 18 (Fig. 1) filled the river, while other locations received untreated domestic sewage. Discharge of domestic sewage into the river from sampling location 4 (Fig. 1) was also observed during the field visit.

Seawater enters the river to a distance of about 1 km from the coast. Sewage and seawater filled the river throughout the year to about 5 km from the sea. When there is no flow in the river in the upper reach from CL to Ramapuram (Fig. 1), the sewage water gets discharged into the river and fills up the small depressions in the river bed and flows. Built-up space occupies most of the area although the western part has vegetation, water bodies, and agriculture. Geologically, at the ground surface in the west and in the river bed, Archean crystalline rocks including granite, gneiss, and charnockite form the basement^22^.

**Figure 2:**
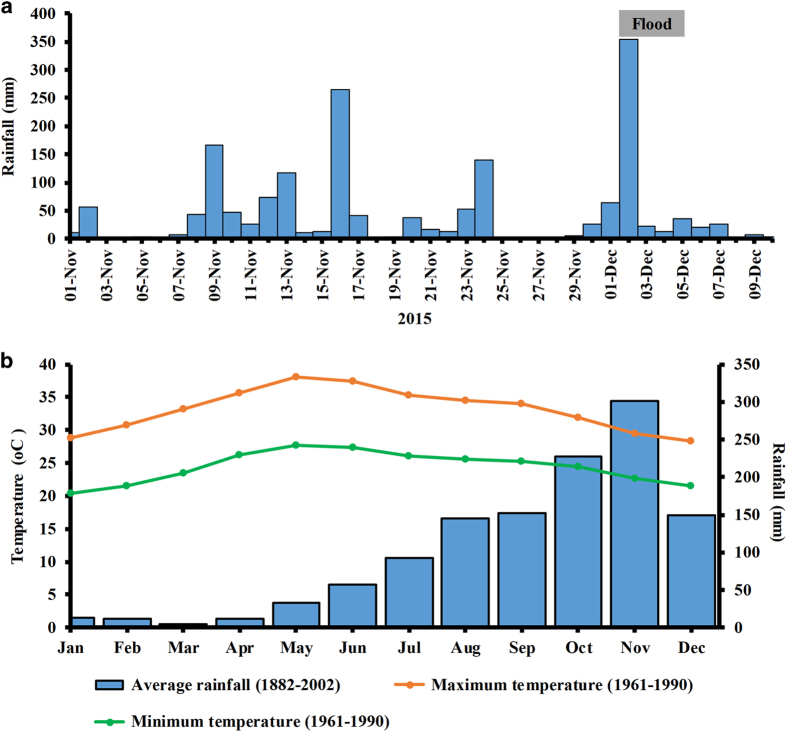
Atmospheric temperature and rainfall in Chennai. Temporal variations in (**a**) daily rainfall leading to flooding from November to December 2015, (**b**) long-term average temperature and rainfall in Chennai.

Chennai received heavy rainfall from 8^th^ to 13^th^ November (475 mm), 265 mm on 16^th^ November and 204 mm from 22^nd^ to 24^th^ November 2015 (ref. 23) (Fig. 2a). In November 2015, the cumulative rainfall was 1,178 mm. Recharge of water caused by rains increased the groundwater head, and AR was flowing at its maximum carrying capacity. During the end of November, the river water level was almost at the rim of the banks. Rainfall for 24 h from 1^st^ December 2015 to 2^nd^ until 8:30 am was 418 mm (Fig. 2a), while, the monthly average during the years 1882 to 2002 was only 300 and 148 mm in November and December, respectively (Fig. 2b). Such an unprecedented heavy rainfall with high intensity on 1^st^ December 2015 resulted in severe flooding on 2^nd^ December in several parts of the city and the AR overflow inundated a large area of about 37.4 km^2^ (Fig. 1) on either side of its banks.

### Hydrogeology

Investigations of large diameter wells during field visits indicated the occurrence of weathered Archean rocks up to ~15 m depth. The top few meters were highly weathered and turned into regolith of thickness varying from 3 to 5 m. A thin layer of alluvium (~3 to 5 m) occurred in some regions along AR, above the Archean rocks and increased to 25 m near the coast. Bore wells were up to 100 m deep as the fractured rock occurred to this depth. Well diameters varied with their purpose: domestic wells (10 cm) and agricultural wells (15 cm). We found wells cased to ~20 m in crystalline rock regions and the casing extended up to a maximum elevation of 60 cm above the surface. Large diameter open wells also exist in this area. Groundwater occurs in weathered/fractured rocks and alluvium in unconfined condition and generally flows eastwards. Groundwater use includes domestic, agricultural and industrial purposes with annual groundwater fluctuation being 20 m^24^. Measured depth to water table was 0–2 m in December 2015 and 2.6–5.5 m during April 2016. We found several inundated wells located on either side of the banks during a flood in December 2015. In all the bore wells, the annular space between the casing and the discharge pipe were not securely protected with water tight seals. This led to the direct entry of surface water into the groundwater zone through the annular space. Apart from this, the flood water also recharged the groundwater zone, whereas during other periods the groundwater table will be much below the river bed.

### Chemical quality

**Figure 3:**
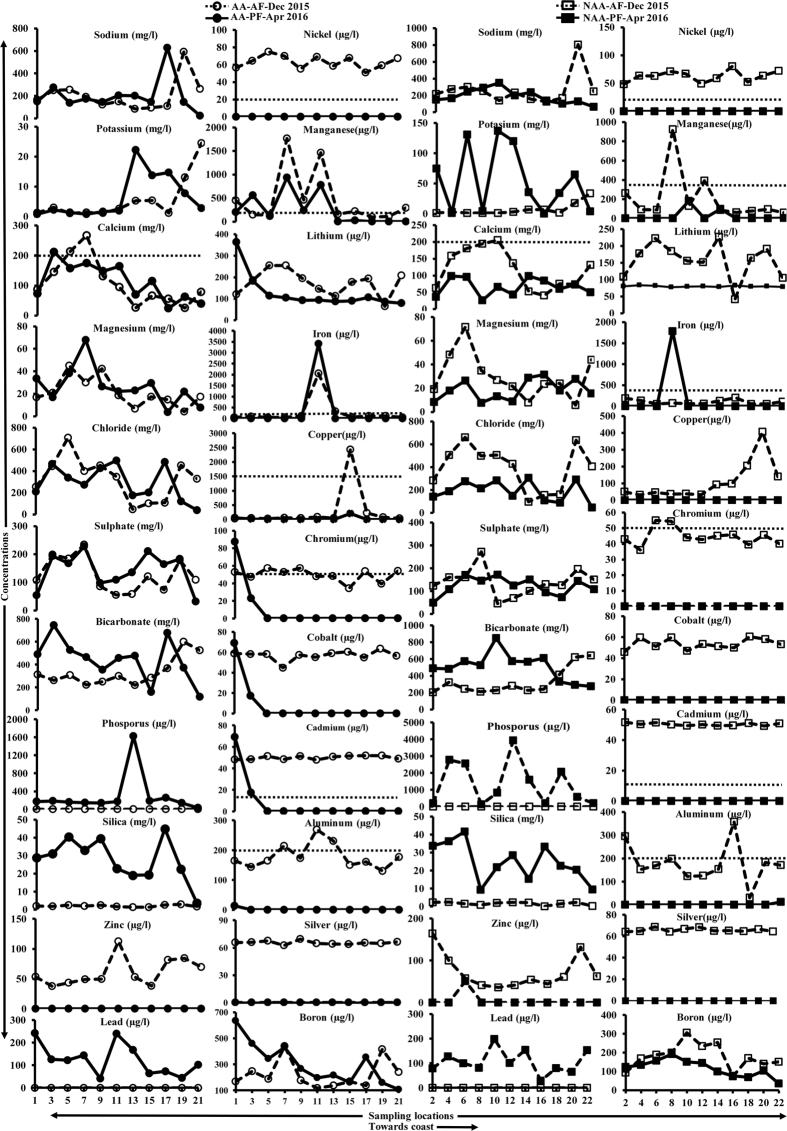
Concentration of ions in groundwater towards the coast (horizontal line refers to the BIS (2012) drinking water limit).

The Stiff pattern shows the general chemical quality of groundwater in affected and non-affected areas during AF at respective locations in Fig. 1. Table 1 gives the summary of minimum and maximum values of measured parameters, the concentration of ions and trace metals as well as bacterial counts in groundwater, AR, and CL. We compared the groundwater quality with drinking water standards specified by the Bureau of Indian Standards (BIS)^25^. pH was in the desirable range (6.5 to 8.5) while, groundwater was unsuitable for direct domestic use in some locations based on TDS>2,000 mg l^−1^ during AF in non-affected areas (Table 1). Concern for water quality based on chemical constituents was mainly due to calcium (>200 mg l^−1^) and sulphate (>400 mg l^−1^) as they exceeded the limits recommended by BIS^25^ (Fig. 3). The concentration of silica was <45 mg l^−1^. Carbonates were not detected in any of the samples. Except for silver, boron, and zinc, the other trace metals were in high concentrations in surface and groundwater (Table 1). Lead content in groundwater during both times of sampling was higher than the maximum permissible limit of 10 μg l^−1^. Nickel and cadmium in groundwater during AF exceeded the limits in all locations, whereas, aluminium exceeded in certain affected locations. High aluminium concentration in locations 7, 11, 13, 2 and 16 reduced to below detectable limits (BDL) during PF. Chromium was near the maximum limit in most affected locations but below the limit in non-affected areas. During AF, groundwater at locations 7 and 11 of affected areas and location 8 of the non-affected region had a high concentration of manganese that remained high during PF in affected areas but reduced in non-affected areas. High iron content observed in locations 11 and 8 increased in PF. AR1, AR2, and CL had desirable pH during both AF and PF. EC was high in downstream (AR2) as expected due to the build-up of ions carried from upstream. CL had desirable EC (<500 μS cm^−1^) as well as major ions which are essential considering the fact that water supply to the city is from this lake. AR2 was higher than AR1 in sodium, potassium, chloride and sulphate concentrations during AF.

In PF, ion concentrations exceeded except for chloride, sulphate, and magnesium (Table 1). This indicated the nature of chemical load/pollutants carried by AR from upstream areas. In CL, the concentration of lead was very high during AF, whereas all other trace elements were much less. Supplementary Fig. 1 indicated no major change in anion concentration (less significant shift in the points vertically), but the concentration of major cations varied reasonably (the points spread out horizontally). Similarly, comparison of plots for AF and PF indicated that although the concentration of major ions varied, their ratio was largely unaltered. Fluctuation in the concentration of ions between wells in affected and non-affected areas also implied that water type was reasonably similar i.e., Na-Cl followed by Ca-Mg-Cl (Supplementary Fig. 1). Overall, the concentration of major ions in groundwater has increased from AF to PF (Fig. 3). The concentration of other elements and trace metals has decreased from AF to PF. We obtained similar results for CL, AR1, and AR2. During the floods, the concentration of trace metals increased significantly indicating contamination from anthropogenic sources of the urbanized environment. There was no major difference in concentration of ions in groundwater of affected and non-affected areas.

In general, the concentration of major ions in groundwater was less during AF, however it increased in PF. In contrast, the concentration of trace metals was higher in AF than PF. This highlights the danger of urban runoff carrying waste such as, domestic sewage, printed papers, metal scraps, e-waste, batteries, oil and paint from service stations that has resulted in an increase of aluminium, cadmium, cobalt, chromium, copper, iron, lithium, manganese, nickel, phosphorus and lead. We attributed decreased concentration of trace metals in PF to sorption processes and reduction in recharge of contaminated urban runoff due to the absence of rainfall.

Boron, silver, and zinc were also found in trace amounts contributed by recharge of the flood waters and decreased to BDL during PF, indicating the reduced quantum of the sources of contamination. The concentration of chromium has increased due to runoff from the eastern side (location 4—having tanning industries). Elevated concentration of chromium in this region has been previously reported^26^. Silica behaved differently with elevated concentration during PF suggesting the role of rockwater interaction due to prolonged residence time, which was in contrast to AF. We witnessed point source contamination as indicated by peaks at certain sampling locations (Fig. 3). Increased concentration of ions (other than silica) during AF than PF (Fig. 3) was due to the enormous quantity of surface runoff entering the contaminated AR from the urban overland flow.

### Bacteriological quality

**Figure 4:**
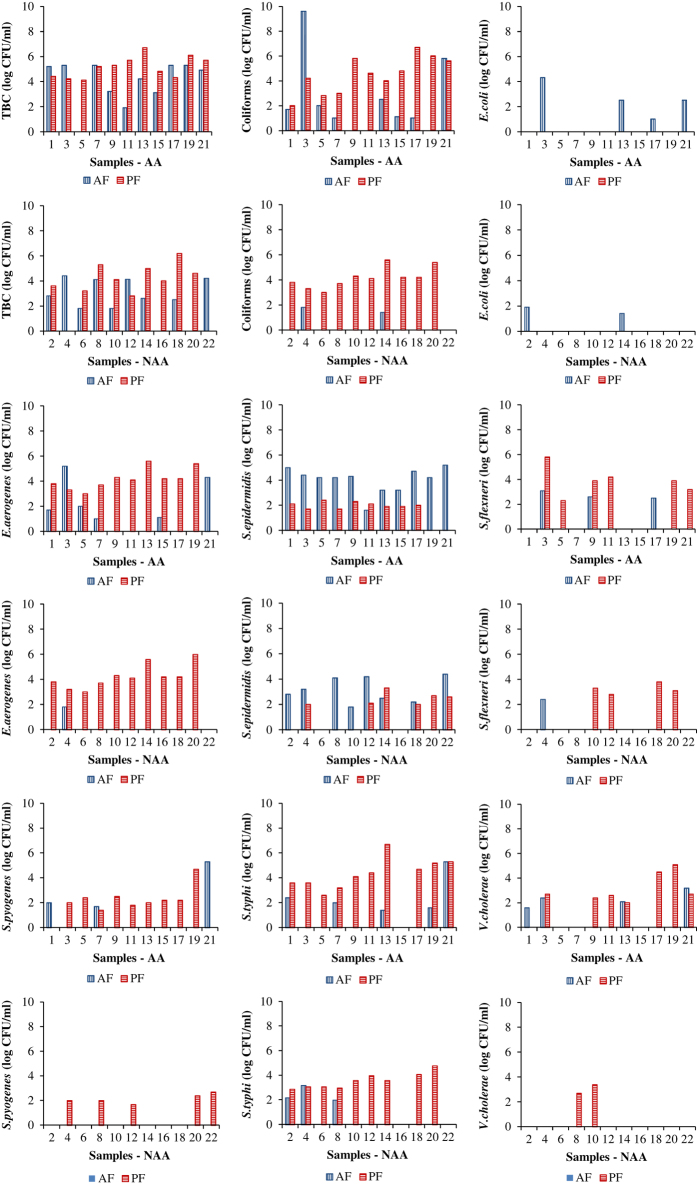
Comparison of TBC and pathogens in water samples during AF and PF in affected and non-affected areas.

Total bacterial count (TBC) in groundwater was high in most affected locations. In non-affected regions, it was comparatively lower and became almost negligible during PF at a few locations. TBC was high in AR1 during AF and increased PF in both AR1 and AR2. As expected, TBC was much lower in CL (Fig. 4). Coliform counts were also found to be high in affected areas but increased during PF in non-affected areas. The count was high in AR2 but reduced during PF while increased in CL. Both *Escherichia coli* and *Enterobacter aerogenes* were high during AF in four affected locations while absent in most non-affected locations. *E.coli* (accession numbers KU981202—KU981210) were absent during PF in both affected and non-affected areas. In contrast, *E.aerogenes* (KU981189—KU981196) was high during PF. We found *E.coli* in AR1 and AR2 during AF but it was absent in PF and CL. *E.aerogenes* was high in AR2 and increased during PF in CL, AR1, and AR2.

*Staphylococcus epidermidis* (KU981229—KU981251) were high during AF in both affected and non-affected areas with more locations of affected areas exposed to the pathogen. The central locations were comparatively less affected. During PF, the pathogen reduced in affected areas but was retained in non-affected locations. It was high in AR2 and increased during PF while absent in CL. We found high counts of *Shigella*, *Streptococcus* and *Salmonella* strains (*S.flexneri*, *S.pyogenes,* and *S.typhi*) in affected areas. These strains proliferated during PF. We observed *S.flexneri* (KU981252—KU981256) in CL, whereas, AR1 and AR2 did not show any sign of its proliferation during AF. We also observed it in PF at both river locations and absent in CL. We found *S.pyogenes* (KU981197—KU981201) and *S.typhi* (KU981211—KU981228) in AR1 and AR2 but not in CL during AF and PF. We observed *Vibrio cholerae* (KU981180—KU981188) only in affected areas during AF and increased in PF. Even though it was completely absent at all non-affected locations in AF, two central locations showed high counts during PF. It was not found in CL but present in AR1 and AR2 during AF and increased in all three locations in PF.

**Figure 5:**
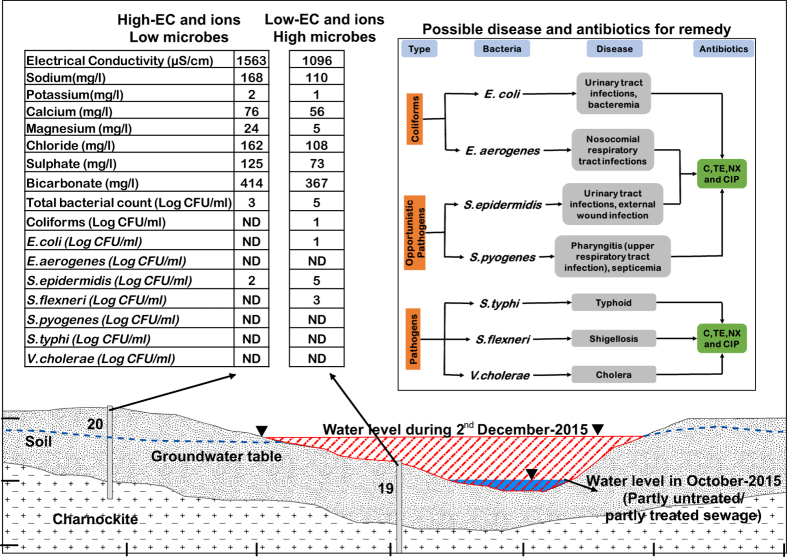
Conceptual diagram with the results at locations 19 and 20 during AF with the possible diseases to humans and antibiotics for remedy.

Our findings indicate that the floods from heavy rainfall influence the numbers and dynamics of TBC, coliforms, and pathogens in surrounding locations. The runoff carrying domestic waste and sewage entered into the contaminated AR and has led to elevated microbial counts in groundwater of affected areas (Fig. 5). Such an observation of high *E.coli* counts in surface water is possibly due to dense clustering of residences with on-site septic systems^27^. *S.pyogenes* and *V.cholerae* observed only in affected areas during AF, signifies the impact of floods in affecting the groundwater quality with enteropathogens. The effect of flooding was evident by observation of coliforms and other pathogens at higher levels in many affected locations. *S.epidermidis* in all the affected locations is an indication of an environment conducive for proliferation in contrast to other pathogens.

Low temperatures, high soil humidity, neutral or alkaline soil pH and the presence of organic carbon enhanced the survival of these microorganisms in soil along with groundwater after the floods^28^. Since this study emphasized on groundwater, soil properties were not analyzed to understand the relationship between these parameters and the bacterial load as carried out by Soupir *et al.*^29^. Elevated counts of TBC, coliforms and other pathogens in all locations during PF is an indicator of favorable conditions for their growth. Further microbial counts have multiplied much more in affected areas indicating the impact of floods even after four months. Among them, high counts of coliforms in groundwater at most affected locations and elevated counts during PF indicated fecal contamination. The cause of the absence of coliforms during AF in CL was due to surface runoff of contaminants to other locations (AR1 and AR2) observed with high coliforms. The cause of significant increase during PF was by natural restoration of an environment favorable for microbial growth. The presence of coliforms in water is not considered suitable for drinking since fecal pollution is an indicator of the pathogenic gastrointestinal bacteria^30^. The absence of coliforms during AF in the lake was due to the reasonably clean catchment. The cause of significant increase during PF was due to the increase in contaminant load from the domestic sewage in AR. Water-borne pathogens considered in this study are of public health significance as they may contribute to disease upon contact.

### Antibiotic susceptibility

Among the seven groups of pathogens found in the water samples, we obtained seventy-seven isolates (Supplementary Table 1) (KU981180—KU981256) by 16S rRNA sequencing and we subjected them to antibiotic susceptibility (Data Citation 1). We grouped antibiotics (Ab) into three categories based on the mode of action namely cell wall inhibition (CTR), protein synthesis inhibition (C, DO and TE) and nucleic acid inhibition (CIP, NX, and NA). Irrespective of affected and non-affected areas during AF and PF, all the seven groups were highly sensitive to C, TE, NX, and CIP. *E.aerogenes* causing nosocomial respiratory tract infections, *E. coli* associated with urinary-tract infections and bacteraemia were susceptible to C, TE, NX and CIP suggesting the effectiveness of these antibiotics for treatment. *E.aerogenes* and *S.pyogenes* were resistant to NA since it was less effective among the antibiotics that target nucleic acid inhibition. *E.aerogenes*, S*.pyogenes,* and *E.coli* showed antibiotic resistance in soil and water environment^31^. These organisms are now increasingly encountered as community-acquired infections^32^.

Environmental changes influence the susceptibility of *Salmonella* and *Shigella* to CTR that targets cell wall inhibition as seen from the increase in sensitivity to the antibiotic during PF in non-affected locations. Increase in sensitivity to CTR as seen in *Salmonella* (typhoid) and *Shigella* (shigellosis-acute bloody diarrhoea) during PF in non-affected areas may be because of competitive inhibition of pathogens as the count increased in PF. *V. cholerae* (causing cholera) was susceptible to all the tested third generation Ab, and we can treat cholera with any of these Ab.

Opportunistic pathogens, *S.epidermidis* (infections associated with intravascular devices, catheters, and external wounds) and *S.pyogenes* (pharyngitis in the upper respiratory tract) were also susceptible to C, TE, NX, and CIP which could serve as therapeutic measures. The task of evaluating the impact of human activities and anthropogenic forces on antibiotic resistance frequencies in water samples is usually a challenge. Differences in composition of species in a habitat appear to dictate the pattern and nature of resistance observed^33^.

### Relation between groundwater chemistry and bacteriological quality

Principal component analysis (PCA) indicated a difference in the measured parameters between affected and non-affected areas (Supplementary Table 2). In affected areas, most of the trace metals were on the positive side of component 1 whereas major ions were on the positive side of component 2. In the case of non-affected locations, major ions, trace metals, and microbial species occurred in the positive and negative side of component 1. Thus, the difference in correlation of major elements and EC indicates the difference in sources between affected and non-affected areas. PCA I exhibited a positive relationship between the ions and the negative relationship among the microbes in affected areas during December 2015. PCA II and III of affected areas were different from non-affected locations during PF and all locations during AF. This shows the complexity of processes that affect the presence of these parameters in groundwater.

There was a significant difference in the relationship among chemical ions and microbes in groundwater of affected and non-affected areas (Supplementary Fig. 2) indicating the impact of floods. In affected areas, most of the trace elements and major ions are in the field of PCA 1>50%, whereas microbes are in the field of PCA<−30% indicating an inverse relationship between them. In PF, PCA 1 involved trace metals and major ions (PCA 1>50%), whereas, PCA 2 showed that microbes influenced the water quality (PCA 2>50%). Thus, PCA analysis indicated that trace metals influenced the groundwater quality during AF, while microbes controlled the water quality in PF.

PCA indicated that both geological and anthropogenic factors influenced the chemical and microbiological quality of groundwater due to flooding. Higher correlation between specific microbial populations with respect to major ions may be due to evolutionary factors such as antibiotic resistance. A previous study reported that the number of strains which exhibited multi-resistance to several Ab and metals was higher in sea/river water samples containing low levels of faecal indicators^34^.

In our study based on groundwater, river water, and lake samples collected from different locations, the isolates of the same species were 99.5% similar in phylogenetic analysis. These results suggest that bacterial strains mostly originated from a single source consisting of domestic sewage, particularly faecal contamination. They were then carried through the flood water, resulting in contamination of groundwater sources. Primarily, there is no baseline data of resistance profiles or patterns in various water habitats before the use of Ab. Also undisturbed control areas may not be absolutely pristine. Antibiotic resistance is common in drinking water^35^.

The present study demonstrated that floods impact the microbial quality of groundwater. Further studies through the year will throw more light on residual effects if any. There was higher microbial contamination and presence of pathogens in affected areas as expected. However, increase in microbial load in both affected and non-affected areas during PF is of special interest. This highlights the impact of a flood event on microbial quality of water for a longer period. These findings emphasise the need for public health intervention to address adverse effects that may result from the poor microbial quality of contaminated water.

At all sampling locations of this densely populated city, groundwater contamination was mainly due to inundation of wells during the flood. Municipal authorities chlorinated most wells and warned the public not to use groundwater for drinking purpose. Further, they provided chlorinated drinking water to the displaced population during the unprecedented floods. After the floods, public health authorities monitored the locations near CL and AR for four months and reported no outbreak of epidemic diseases except for a few reported cases of diarrhoea or typhoid illness. They advised the population living in coastal areas against the use of wellwater for drinking purpose. Although we cannot prevent such extreme climatic events, we can minimise the extent of adverse impact through better awareness. The present study on Chennai floods can minimise the impact of such events in future by control of vector-borne diseases.

## Usage Notes

The dataset includes EC, pH, dissolved oxygen, Ca, Mg, Na, K, Cl, SO_4_, HCO_3_, Si, Ag, Al, B, Cd, Co, Cr, Cu, Fe, Li, Mn, Ni, Pb, Zn, P, total bacterial count, coliforms, *E.coli, E.aerogenes, S.epidermidis, S.flexneri, S.pyogenes, S.typhi* and *V.cholerae* of surface water and groundwater collected after an extreme flood event in Chennai, a large metropolitan city in India. Genotype data in GenBank gives sequence information (16S rRNA) for each pathogen isolated from the water samples. Further, the antibiotic susceptibility of pathogens to use in the event of potential contact with water by humans is also given in a separate table. These datasets together present the first extensive spatiotemporal assessment of chemical and microbial contents of surface and groundwater after a major flood event. This research emphasises the antibiotic susceptibility of pathogens encountered during such calamities as a precautionary measure. The antibiotic susceptibility of pathogens will help to improve the treatment of severe cases affected by contact with contaminated water through an appropriate selection of prescribed medication henceforth. The datasets serve a basis for understanding the antibiotic susceptibility of past and future flood events in the outbreak of epidemic diseases.

## Additional information

**How to cite this article:** Gowrisankar, G. *et al.* Chemical, microbial and antibiotic susceptibility analyses of groundwater after a major flood event in Chennai. *Sci. Data* 4:170135 doi: 10.1038/sdata.2017.135 (2017).

**Publisher’s note:** Springer Nature remains neutral with regard to jurisdictional claims in published maps and institutional affiliations.

## Supplementary Material



Supplementary Figures

Supplementary Table 1

Supplementary Table 2

## Figures and Tables

**Table 1:** Summary of geochemical analyses of water samples.

**Parameters**	**Drinking water limit (BIS 2012)**	**Groundwater**		**Surface water**											
		**Dec 2015—AA (AF)**		**Apr 2016—AA (PF)**		**Dec 2015—NAA (AF)**		**Apr 2016—NAA(PF)**		**December 2015 (AF)**			**April 2016 (PF)**		
		**Min**	**Max**	**Min**	**Max**	**Min**	**Max**	**Min**	**Max**	**AR1**	**AR2**	**CL**	**AR1**	**AR2**	**CL**
pH	6.5–8.5	6.7	8.0	6.4	7.9	6.9	8.4	6.5	7.5	7.9	7.8	7.8	7.1	7.1	8.0
Total Dissolved Solids (mg l^−1^) derived from Electrical Conductivity	500–2000	408	1915	228	1976	515	2503	458	1748	1156	1306	194	382	1820	172
*Major ions (mg l^−1^)*															
Calcium	75–200	25	267	24	212	41	205	26	98	137	45	18	26	44	29
Magnesium	30–100	4	45	4	68	6	72	8	32	55	37	7	16	14	8
Sodium	Nil	84	592	25	629	125	805	64	350	227	281	22	64	406	18
Potassium	NIl	1.1	24.5	0.8	40.8	1.2	33.8	0.3	137.0	9.5	14.8	6.3	10.2	27.3	3.4
Bicarbonate	Nil	219	600	116	921	205	641	275	848	464	256	86	195	372	131
Chloride	250–1000	46	709	41	500	96	661	47	307	341	547	20	73	547	20
Sulphate	200–400	55	234	14	228	46	272	50	172	25	96	10	37	84	13
*Minor ions*															
Silica (mg l^−1^)	Nil	1.3	2.9	3.6	44.9	0.1	2.5	9.4	41.7	0.9	0.7	0.3	6.8	13.4	3.4
Silver (μg l^−1^)	100	62.6	69.4	BDL	BDL	64.1	68.7	BDL	BDL	63.6	64.2	66.0	BDL	BDL	BDL
Aluminium (μg l^−1^)	30–200	130.8	268.9	BDL	BDL	31.0	356.9	BDL	11.6	271.1	246.4	230.5	8.5	162.8	BDL
Boron (μg l^−1^)	500–1000	118.0	438.6	106.5	460.1	73.1	305.0	35.5	191.5	158.2	160.7	90.2	119.4	185.6	19.6
Cadmium (μg l^−1^)	30	47.9	51.9	BDL	69.0	49.2	51.7	BDL	BDL	47.9	51.9	50.4	154.6	163.8	BDL
Cobalt (μg l^−1^)	Nil	44.7	63.5	BDL	75.2	45.9	60.4	BDL	BDL	58.0	60.0	47.0	154.6	78.8	BDL
Chromium (μg l^−1^)	50	34.0	57.3	BDL	87.5	36.1	54.9	BDL	BDL	67.2	58.0	47.7	BDL	BDL	BDL
Copper (μg l^−1^)	50–1500	27.6	2430.5	BDL	203.2	31.1	406.6	BDL	BDL	93.8	63.2	84.1	6.5	5.4	BDL
Iron (μg l^−1^)	300	55.8	2061.0	BDL	3416.0	56.7	207.7	BDL	1788.7	336.7	402.2	253.0	448.4	526.2	BDL
Lithium (μg l^−1^)	Nil	65.5	255.0	79.2	363.6	41.1	226.8	77.8	83.4	83.3	190.4	97.6	58.6	209.8	77.4
Manganese (μg l^−1^)	100–300	95.7	1762.7	BDL	932.4	61.3	925.6	BDL	181.3	407.2	347.5	75.1	578.5	376.6	BDL
Nickel (μg l^−1^)	20	51.1	74.8	BDL	BDL	48.3	80.2	BDL	BDL	38.8	58.1	205.4	BDL	BDL	BDL
Lead (μg l^−1^)	10	BDL	69.1	42.2	243.3	BDL	BDL	29.0	197.8	38.8	58.1	205.4	BDL	BDL	64.2
Zinc (μg l^−1^)	5000–15000	37.6	112.5	BDL	BDL	36.4	164.1	BDL	51.4	102.0	48.6	51.2	BDL	BDL	64.2
Phosphorus (μg l^−1^)	Nil	BDL	BDL	30.2	1625.0	BDL	BDL	122.3	3936.8	BDL	BDL	BDL	175.0	196.4	101.1
*Microorganisms (log CFU ml^−1^)*															
Total Bacterial Count	Nil	ND	5.3	4.1	6.7	ND	4.4	ND	6.2	5.2	4.9	3.2	7.2	6.4	2.2
Coliforms	Nil	ND	9.6	2	6.7	ND	1.8	ND	5.6	5.2	6.8	ND	4.1	5.3	2.8
*E.coli*	Nil	ND	4.3	ND	ND	ND	1.9	ND	ND	2.9	2.5	ND	ND	ND	ND
*E.aerogenes*	Nil	ND	5.2	ND	5.6	ND	1.8	ND	6	2.2	4.3	2.5	4.1	5.3	2.8
*S.epidermidis*	Nil	1.6	5.2	ND	2.4	ND	4.4	ND	3.3	4.1	5.2	ND	6.3	6	ND
*S.flexneri*	Nil	ND	3.1	ND	5.8	ND	2.4	ND	3.8	ND	ND	3.2	1.1	1	ND
*S.pyogenes*	Nil	ND	5.3	ND	4.7	ND	ND	ND	2.7	2.5	5.3	ND	3.9	5.3	ND
*S.typhi*	Nil	ND	5.3	ND	6.7	ND	3.2	ND	4.8	5.3	5.3	ND	5.7	6.8	ND
*V.cholerae*	Nil	ND	3.2	ND	5.1	ND	ND	ND	3.4	2.9	3.2	ND	4.12	5.39	1.84
ND—Not Detected, Electrical Conductivity (μs cm^−1^), BDL—Below Detectable Limit, Nil—No limit permissible by BIS (2012).															

## References

[d1] Figshare Gowrisankar G. 2017 https://doi.org/10.6084/m9.figshare.c.3854080.v1

[d2] GenBank Ramachandran C. 2016 KU981180.1—KU981256.1

