# Supplementary material for: Chemical, microbial and antibiotic susceptibility analyses of groundwater after a major flood event in Chennai
Source: Sci Data. 2017 Oct 10;4:170135. doi: 10.1038/sdata.2017.135 (PMC5634326; doi:10.1038/sdata.2017.135)
Supplement: Supplementary Figures [file sdata2017135-s2.pdf]

TABLE OF CONTENTS

Title	Page no.
Supplementary Figure 1 Chadha diagram depicting the water types based on major ion concentrations	2
Supplementary Figure 2 Cross-plot of the PCA for hydrochemical components (a) affected areas during AF (Dec 2015) and PF (Apr 2016), (b) non-affected areas during AF and PF	3

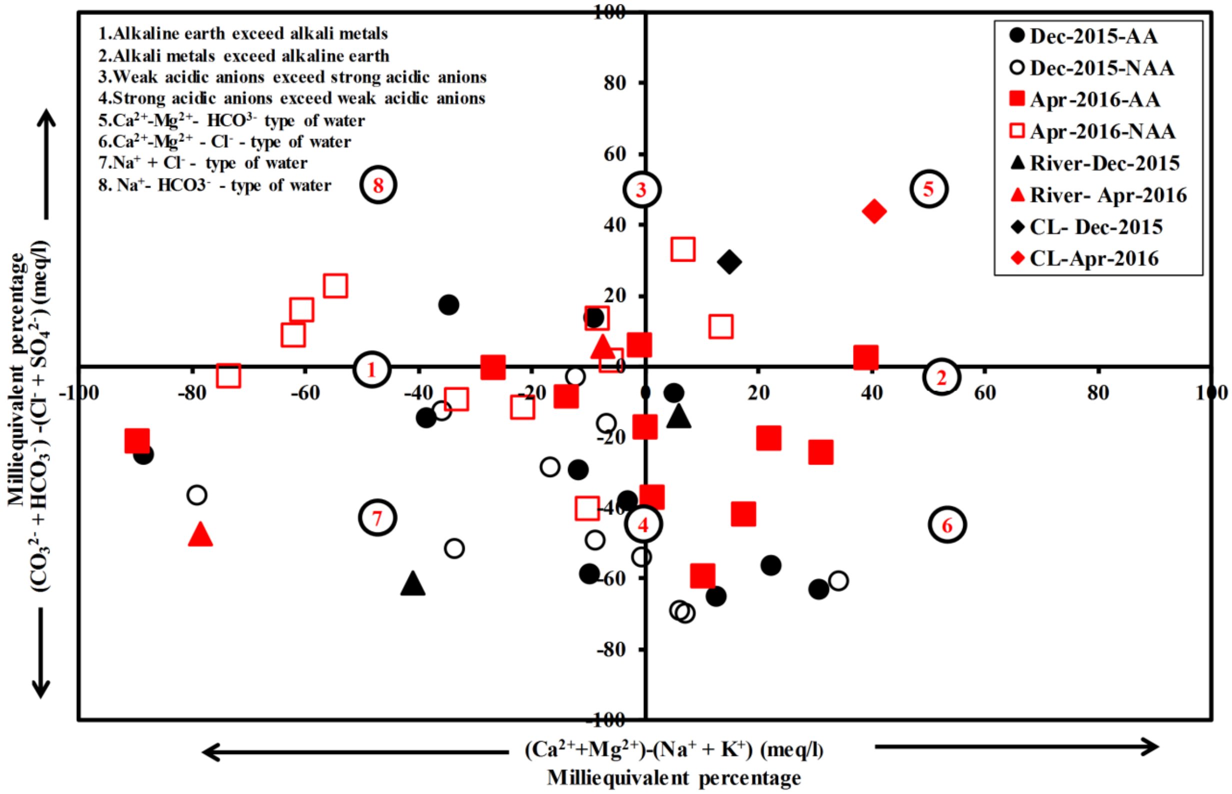

Supplementary Figure 1 Chadha diagram depicting the water types based on major ion concentrations

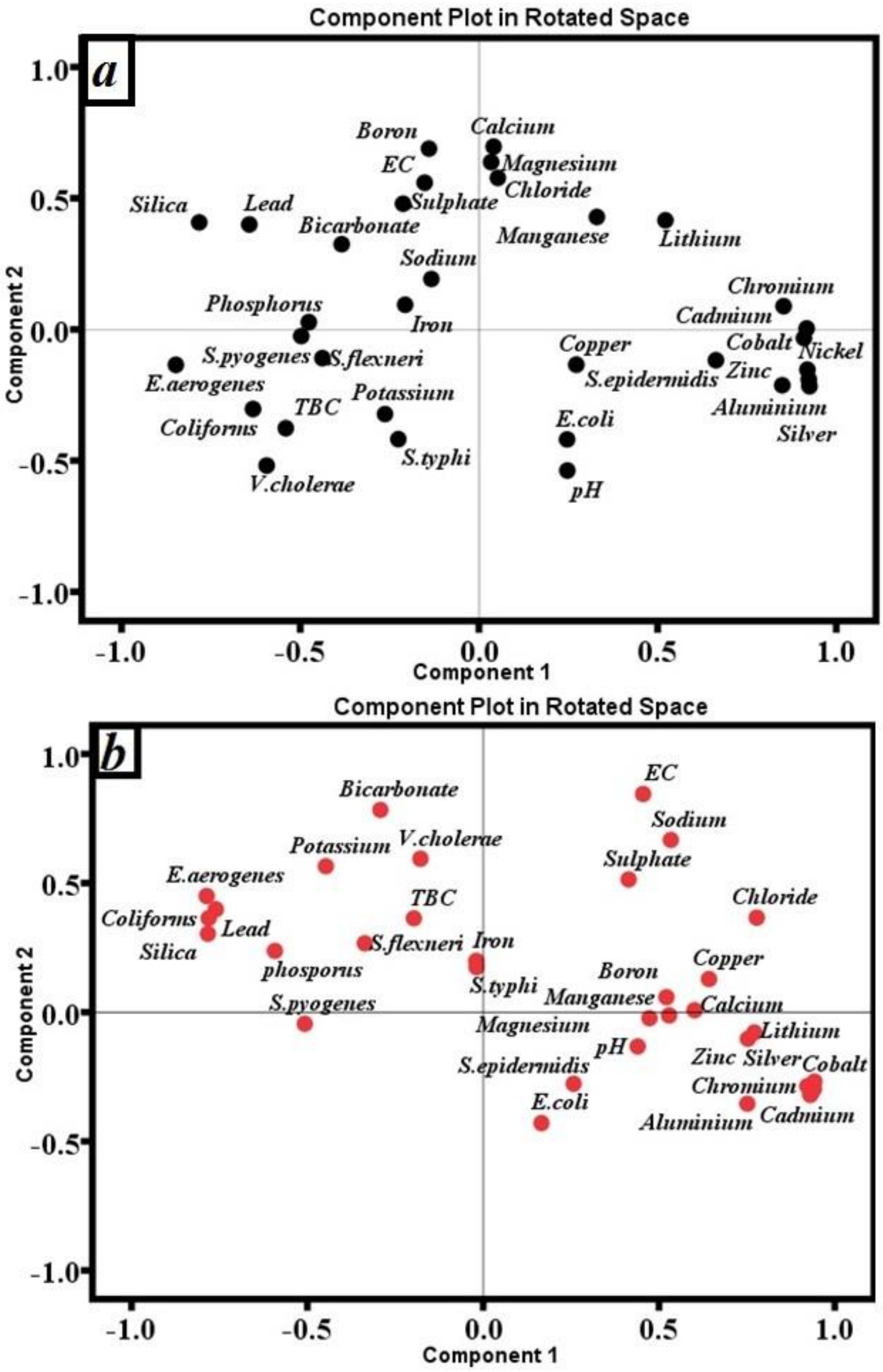

Supplementary Figure 2 Cross-plot of the PCA for hydrochemical components (a) affected areas during AF (Dec 2015) and PF (Apr 2016), (b) non-affected areas during AF and PF
