# Supplementary material for: Chemical, microbial and antibiotic susceptibility analyses of groundwater after a major flood event in Chennai
Source: Sci Data. 2017 Oct 10;4:170135. doi: 10.1038/sdata.2017.135 (PMC5634326; doi:10.1038/sdata.2017.135)
Supplement: Supplementary Table 1 [file sdata2017135-s3.docx]

|  | **Multiple Sequence Alignment (MAFFT)** |
| --- | --- |
| ***Escherichia coli*** | 1_seq23__Organism=_Escherichia_coli___Strain_UERSG23__16S_rRNA_sequence_Isolate_from_Tube_well_water:0.00450,  4_seq26__Organism=_Escherichia_coli___Strain_UERSG26__16S_rRNA_sequence_Isolate_from_Tube_well_water:0.00450):0.00500,  3_seq25__Organism=_Escherichia_coli___Strain_UERSG25__16S_rRNA_sequence_Isolate_from_Tube_well_water:0.00950):0.00050,  5_seq27__Organism=_Escherichia_coli___Strain_UERSG27__16S_rRNA_sequence_Isolate_from_Tube_well_water:0.01000):0.00155,  9_seq31__Organism=_Escherichia_coli___Strain_UERSG31__16S_rRNA_sequence_Isolate_from_Tube_well_water:0.01155):0.00185,  6_seq28__Organism=_Escherichia_coli___Strain_UERSG28__16S_rRNA_sequence_Isolate_from_Tube_well_water:0.01340):0.00243,  7_seq29__Organism=_Escherichia_coli___Strain‐_UERSG29__16S_rRNA_sequence_Isolate_from_Tube_well_water:0.01050,  8_seq30__Organism=_Escherichia_coli___Strain_UERSG30__16S_rRNA_sequence_Isolate_from_Tube_well_water:0.01050):0.00533):0.08397,  2_seq24__Organism=_Escherichia_coli___Strain‐_UERSG24__16S_rRNA_sequence_Isolate_from_Tube_well_water:0.09980) |
| ***Enterobacter aerogenes*** | 1_Seq10__Organism=_Enterobacter_aerogenes___Strain‐_UERSG10__16S_rRNA_sequence_Isolate_from_Tube_well_water:0.00600,  2_Seq11__Organism=_Enterobacter_aerogenes___Strain‐_UERSG11__16S_rRNA_sequence_Isolate_from_Tube_well_water:0.00600):0.00205,  3_Seq12__Organism=_Enterobacter_aerogenes___Strain‐_UERSG12__16S_rRNA_sequence_Isolate_from_Tube_well_water:0.00805):0.00822,  5_Seq14__Organism=_Enterobacter_aerogenes___Strain‐_UERSG14__16S_rRNA_sequence_Isolate_from_Tube_well_water:0.01577  6_Seq15__Organism=_Enterobacter_aerogenes___Strain‐_UERSG15__16S_rRNA_sequence_Isolate_from_Tube_well_water:0.00600,  8_Seq17__Organism=_Enterobacter_aerogenes___Strain‐_UERSG17__16S_rRNA_sequence_Isolate_from_Tube_well_water:0.00600):0.00050,  7_Seq16__Organism=_Enterobacter_aerogenes___Strain_UERSG16__16S_rRNA_sequence_Isolate_from_Tube_well_water:0.00650):0.00927):0.00050):0.13316,  4_Seq13__Organism=_Enterobacter_aerogenes___Strain‐_UERSG13__16S_rRNA_sequence_Isolate_from_Tube_well_water:0.14943) |
| ***Salmonella typhi*** | 1_seq32__Organism=_Salmonella_typhi___Strain‐_UERSG32__16S_rRNA_sequence_Isolate_from_Tube_well_water_:0.00600,  7_seq38__Organism=_Salmonella_typhi___Strain‐_UERSG38__16S_rRNA_sequence_Isolate_from_Tube_well_water_:0.00600):0.00005,  3_seq34__Organism=_Salmonella_typhi___Strain‐_UERSG34__16S_rRNA_sequence_Isolate_from_Tube_well_water_:0.00350,  5_seq36__Organism=_Salmonella_typhi___Strain_UERSG36__16S_rRNA_sequence_Isolate_from_Tube_well_water_:0.00350):0.00255):0.00063  13_seq44__Organism=_Salmonella_typhi___Strain‐_UERSG44__16S_rRNA_sequence_Isolate_from_Tube_well_water_:0.00668):0.00100,  9_seq40__Organism=_Salmonella_typhi___Strain‐_UERSG40__16S_rRNA_sequence_Isolate_from_Tube_well_water_:0.00650,  11_seq42__Organism=_Salmonella_typhi___Strain‐_UERSG42__16S_rRNA_sequence_Isolate_from_Tube_well_water_:0.00650):0.00050,  17_seq48__Organism=_Salmonella_typhi___Strain‐_UERSG48__16S_rRNA_sequence_Isolate_from_Tube_well_water_:0.00700):0.00001,  15_seq46__Organism=_Salmonella_typhi___Strain_UERSG46__16S_rRNA_sequence_Isolate_from_Tube_well_water_:0.00701):0.00067):0.  2_seq33__Organism=_Salmonella_typhi___Strain‐_UERSG33__16S_rRNA_sequence_Isolate_from_Tube_well_water_:0.00950,  6_seq37__Organism=_Salmonella_typhi___Strain_UERSG37__16S_rRNA_sequence_Isolate_from_Tube_well_water_:0.00950):0.00156):0.00220  4_seq35__Organism=_Salmonella_typhi___Strain‐_UERSG35__16S_rRNA_sequence_Isolate_from_Tube_well_water_:0.00600,  8_seq39__Organism=_Salmonella_typhi___Strain‐_UERSG39__16S_rRNA_sequence_Isolate_from_Tube_well_water_:0.00600):0.00045,  12_seq43__Organism=_Salmonella_typhi___Strain‐_UERSG43__16S_rRNA_sequence_Isolate_from_Tube_well_water_:0.00645):0.00005,  14_seq45__Organism=_Salmonella_typhi___Strain‐_UERSG45__16S_rRNA_sequence_Isolate_from_Tube_well_water_:0.00650):0.00153,(  16_seq47__Organism=_Salmonella_typhi___Strain‐_UERSG47__16S_rRNA_sequence_Isolate_from_Tube_well_water_:0.00800,  18_seq49__Organism=_Salmonella_typhi___Strain_UERSG49__16S_rRNA_sequence_Isolate_from_Tube_well_water_:0.00800):0.00003)  10_seq41__Organism=_Salmonella_typhi___Strain‐_UERSG41__16S_rRNA_sequence_Isolate_from_Tube_well_water_:0.07344) |
| ***Staphylococcus epidermidis*** | 1_Seq50__Organism=_Staphylococcus_epidermidis__Strain‐_UERSG50__16S_rRNA_sequence_Isolate_from_Tube_well_water_:0.00850,  12_Seq61__Organism=_Staphylococcus_epidermidis__Strain‐_UERSG51__16S_rRNA_sequence_Isolate_from_Tube_well_water_:0.00850):0.00068,  4_Seq53__Organism=_Staphylococcus_epidermidis__Strain‐_UERSG53__16S_rRNA_sequence_Isolate_from_Tube_well_water_:0.00850,  17_Seq66__Organism=_Staphylococcus_epidermidis__Strain‐_UERSG66__16S_rRNA_sequence_Isolate_from_Tube_well_water_:0.00850):0.00068):0.00032,  8_Seq57__Organism=_Staphylococcus_epidermidis__Strain‐_UERSG57__16S_rRNA_sequence_Isolate_from_Tube_well_water_:0.00950):0.00150,  14_Seq63__Organism=_Staphylococcus_epidermidis__Strain‐_UERSG63__16S_rRNA_sequence_Isolate_from_Tube_well_water_:0.01100):0.00033,  2_Seq51__Organism=_Staphylococcus_epidermidis__Strain‐_UERSG51__16S_rRNA_sequence_Isolate_from_Tube_well_water_:0.00850,  23_Seq72__Organism=_Staphylococcus_epidermidis__Strain‐_UERSG72__16S_rRNA_sequence_Isolate_from_Tube_well_water_:0.00850):0.00050,  18_Seq67__Organism=_Staphylococcus_epidermidis__Strain‐_UERSG67__16S_rRNA_sequence_Isolate_from_Tube_well_water_:0.00900):0.00100,  6_Seq55__Organism=_Staphylococcus_epidermidis__Strain‐_UERSG55__16S_rRNA_sequence_Isolate_from_Tube_well_water_:0.01000):0.00053,(  3_Seq52__Organism=_Staphylococcus_epidermidis__Strain‐_UERSG52__16S_rRNA_sequence_Isolate_from_Tube_well_water_:0.01006,(((  5_Seq54__Organism=_Staphylococcus_epidermidis__Strain‐_UERSG54__16S_rRNA_sequence_Isolate_from_Tube_well_water_:0.00900,  10_Seq59__Organism=_Staphylococcus_epidermidis__Strain‐_UERSG51__16S_rRNA_sequence_Isolate_from_Tube_well_water_:0.00900):0.00000,  15_Seq64__Organism=_Staphylococcus_epidermidis__Strain‐_UERSG51__16S_rRNA_sequence_Isolate_from_Tube_well_water_:0.00900):0.00021,  21_Seq70__Organism=_Staphylococcus_epidermidis__Strain‐_UERSG70__16S_rRNA_sequence_Isolate_from_Tube_well_water_:0.00920):0.00086):0.00047):0.00047,  11_Seq60__Organism=_Staphylococcus_epidermidis__Strain‐_UERSG51__16S_rRNA_sequence_Isolate_from_Tube_well_water_:0.01100):0.00033):0.00033,  13_Seq62__Organism=_Staphylococcus_epidermidis__Strain‐_UERSG51__16S_rRNA_sequence_Isolate_from_Tube_well_water_:0.01167):0.00040,  7_Seq56__Organism=_Staphylococcus_epidermidis__Strain‐_UERSG56__16S_rRNA_sequence_Isolate_from_Tube_well_water_:0.00550,  9_Seq58__Organism=_Staphylococcus_epidermidis__Strain‐_UERSG58__16S_rRNA_sequence_Isolate_from_Tube_well_water_:0.00550):0.00656):0.00094,  22_Seq71__Organism=_Staphylococcus_epidermidis__Strain‐_UERSG71__16S_rRNA_sequence_Isolate_from_Tube_well_water_ :0.01300):0.00322,(  16_Seq65__Organism=_Staphylococcus_epidermidis__Strain‐_UERSG65__16S_rRNA_sequence_Isolate_from_Tube_well_water_ :0.01050,  20_Seq69__Organism=_Staphylococcus_epidermidis__Strain‐_UERSG69__16S_rRNA_sequence_Isolate_from_Tube_well_water_ :0.01050):0.00572):0.10866,  19_Seq68__Organism=_Staphylococcus_epidermidis__Strain‐_UERSG68__16S_rRNA_sequence_Isolate_from_Tube_well_water_ :0.12488) |
| ***Shigella flexneri*** | 1_Seq73__Organism=_Shigella_flexneri___Strain‐_UERSG73__16S_rRNA_sequence_Isolate_from_Tube_well_water_:0.00650,  5_Seq77__Organism=_Shigella_flexneri___Strain‐_UERSG77__16S_rRNA_sequence_Isolate_from_Tube_well_water_:0.00650):0.00120,  3_Seq75__Organism=_Shigella_flexneri___Strain‐_UERSG75__16S_rRNA_sequence_Isolate_from_Tube_well_water_:0.00770):0.08949,(  2_Seq74__Organism=_Shigella_flexneri___Strain‐_UERSG74__16S_rRNA_sequence_Isolate_from_Tube_well_water_:0.01200,  4_Seq76__Organism=_Shigella_flexneri___Strain‐_UERSG76__16S_rRNA_sequence_Isolate_from_Tube_well_water_:0.01200):0.08519) |
| ***Streptococcus pyogenes*** | 1_Seq18__Organism=_Streptococcus_pyogenes___Strain‐_UERSG18__16S_rRNA_sequence_Isolate_from_Tube_well_water :0.00000,  3_Seq20__Organism=_Streptococcus_pyogenes___Strain‐_UERSG20__16S_rRNA_sequence_Isolate_from_Tube_well_water :0.00000):0.00700,  5_Seq22__Organism=_Streptococcus_pyogenes___Strain‐_UERSG22__16S_rRNA_sequence_Isolate_from_Tube_well_water :0.00700):0.11384,  2_Seq19__Organism=_Streptococcus_pyogenes___Strain‐_UERSG19__16S_rRNA_sequence_Isolate_from_Tube_well_water :0.00650,  4_Seq21__Organism=_Streptococcus_pyogenes___Strain‐_UERSG21__16S_rRNA_sequence_Isolate_from_Tube_well_water :0.00650):0.11434) |
| ***Vibrio cholerae*** | 1_seq1__Organism=_Vibrio_cholerae___Strain‐_UERSG1__16S_rRNA_sequence_____Isolate_from_Tube_well_water:0.00550,  9_seq9__Organism=_Vibrio_cholerae___Strain‐_UERSG9__16S_rRNA_sequence_____Isolate_from_Tube_well_water:0.00550):0.00050,(  3_seq3__Organism=_Vibrio_cholerae___Strain‐_UERSG3__16S_rRNA_sequence_____Isolate_from_Tube_well_water:0.00300,  5_seq5__Organism=_Vibrio_cholerae___Strain‐_UERSG5__16S_rRNA_sequence_____Isolate_from_Tube_well_water:0.00300):0.00300):0.00044,  7_seq7__Organism=_Vibrio_cholerae___Strain‐_UERSG7__16S_rRNA_sequence_____Isolate_from_Tube_well_water:0.00644):0.00308,  2_seq2__Organism=_Vibrio_cholerae___Strain‐_UERSG2__16S_rRNA_sequence_____Isolate_from_Tube_well_water:0.00600,  4_seq4__Organism=_Vibrio_cholerae___Strain‐_UERSG4__16S_rRNA_sequence_____Isolate_from_Tube_well_water:0.00600):0.00050,  8_seq8__Organism=_Vibrio_cholerae___Strain‐_UERSG8__16S_rRNA_sequence_____Isolate_from_Tube_well_water:0.00650):0.00301):0.02505,  6_seq6__Organism=_Vibrio_cholerae___Strain‐_UERSG6__16S_rRNA_sequence_____Isolate_from_Tube_well_water:0.03456) |
