# Supplementary material for: Chemical, microbial and antibiotic susceptibility analyses of groundwater after a major flood event in Chennai
Source: Sci Data. 2017 Oct 10;4:170135. doi: 10.1038/sdata.2017.135 (PMC5634326; doi:10.1038/sdata.2017.135)
Supplement: Supplementary Table 2 [file sdata2017135-s4.docx]

| Sampling time | Principal components | | | | | |
| --- | --- | --- | --- | --- | --- | --- |
|  | I | | II | | III | |
|  | Positive | Negative | Positive | Negative | Positive | Negative |
| Dec-2015-AA | Calcium, Magnesium, Chloride, Sulfate, Lithium, Manganese, Nickel | pH, Cobalt | Coliforms, *E.coli*, *E.aerogenes, S.progenes, S.typi, V.cholerae* | ND | Electrical Conductivity, Sodium, Chloride, Sulfate, Bicarbonate, *S.epidermidis*, Silica | Aluminium, Iron |
| Dec-2015-NAA | Calcium, Chloride, Silica, Boron | *E.coli*, Aluminium, Iron, Lithium | Electrical Conductivity, Sodium, Potassium, Bicarbonate, Silica, Cobalt, Copper | ND | pH, Total Bacterial Count, *E.coli, S.epidermidis* | Silver |
| April-2016-AA | Aluminium, Boron, Cadmium, Cobalt, Chromium, Lithium, Lead | *S.pyogenes* | pH, Total Bacterial Count, *E.coli, E.aerogene, S.typi, V.cholerae* | Magnesium | Electrical Conductivity, Sodium, Chloride, Bicarbonate, *S.epidermidis, S.pyogenes, V.cholerae* | ND |
| April-2016-NAA | Electrical Conductivity, Sodium, Chloride, Sulfate, Bicarbonate, *V.cholerae*, Boron, Manganese, Lead | ND | Total Bacterial Count, Coliforms, *E.aerogenes* | Aluminium | Calcium, Magnesium, Silica, Lithium | *S.typi, V.cholerae*, Iron |

*ND - Not Detected
